# Comprehensive evaluation of drought tolerance of six Chinese chestnut varieties (clones) based on flavonoids and other physiological indexes

**DOI:** 10.1038/s41598-024-65479-2

**Published:** 2024-06-24

**Authors:** Yangjuan Zeng, Chunlei Guo, Meng Wang, Junting Jin, Keyan Yu, Jingzheng Zhang, Fei Cao

**Affiliations:** 1 College of Horticulture Science and Technology, Hebei Normal University of Science and Technology, Changli, 066600 China; 2 Engineering Research Center of Chestnut Industry Technology, Ministry of Education, Qinhuangdao, 066004 China; 3 Hebei Key Laboratory of Horticultural Germplasm Excavation and Innovative Utilization, Qinhuangdao, 066004 China; 4 Hebei Collaborative Innovation Center of Chestnut Industry, Qinhuangdao, 066004 China

**Keywords:** Chestnut, Comprehensive evaluation of drought tolerance, Drought stress, Principal component analysis, Subordinate function, Plant sciences, Plant ecology, Plant physiology, Plant stress responses

## Abstract

Flavonoids are crucial secondary metabolites that possess the ability to mitigate UV damage and withstand both biotic and abiotic stresses. Therefore, it is of immense significance to investigate the flavonoid content as a pivotal indicator for a comprehensive assessment of chestnut's drought tolerance. This study aimed to determine the flavonoid content and drought tolerance-related physiological and biochemical indices of six chestnut varieties (clones) grafted trees—Qianxi 42 (QX42), Qinglong 45 (QL45), Yanshanzaofeng (YSZF), Yanzi (YZ), Yanqiu (YQ), and Yanlong (YL)-under natural drought stress. The results were used to comprehensively analyze the drought tolerance ability of these varieties. The study revealed that the ranking of drought tolerance indices in terms of their ability to reflect drought tolerance was as follows: superoxide (oxide) dismutase (SOD) activity, ascorbate peroxidase (APX) activity, flavone content, catalase (CAT) activity, proline (PRO) content, soluble sugar content, peroxidase (POD) activity, betaine content, flavonol content, hydrogen peroxide (H_2_O_2_) content, soluble protein content, superoxide ion (OFR) content, superoxide (ion OFR) production rate, malondialdehyde (MDA) content, chlorophyll content. Through principal component analysis, the contents of flavonoids and flavonols can be used as indicators for comprehensive evaluation of drought tolerance of chestnut. The comprehensive evaluation order of drought tolerance of grafted trees of 6 chestnut varieties (Clones) was: QL45 > QX42 > YQ > YZ > YSZF > YL.

## Introduction

Chestnut (*Castanea mollissima* Blume), belonging to *Castanea mollissima*, has a long cultivation history and is an economically important fruit tree species with wide distribution, diverse processing products and high comprehensive utilization value^1^. The fruit industry across the world is facing a major constraint in the form of drought due to climate change. This has led to a significant hindrance in its development^2,3^. Accurate evaluation of drought tolerance of chestnut is very important for selecting cultivars and improving the yield and quality of chestnut^4^. Water determines the species and quantity of plants^5,6^. Changes in the physiological responses of plants to drought tolerance have been extensively studied such as changes in physiological indices, e.g., root morphology, osmotic adjustment and antioxidant enzyme activity^7–10^. When plants are subjected to drought stress, leaves are most sensitive to drought. Under drought conditions, water transport is slow or even stops, which hinders photosynthesis and affects plant survival and development. Chlorophyll, leaf relative water content and nutrient content stored are significantly reduced, and finally, the yield is reduced^11^. Generally, the osmotic substances in leaves significantly increase with the deepening of drought such as the accumulation of soluble sugar, proline (PRO) and betaine. Osmotic regulation can help plants stabilize cell expansion, maintain normal photosynthesis, protect enzyme activity and improve drought tolerance^12^.

Under drought stress, the antioxidant system in plants also responds to drought stress in a timely manner, improving the activities of related enzymes such as superoxide dismutase (SOD) and peroxidase (POD) to remove harmful substances produced by drought in plants and reducing the impact of stress on the normal life activities of plants^13^. Abiotic stress likes drought or salinity concurrently causes oxidative stress^14^ and osmotic stress^15^ which affect the biochemical and physiological processes of plants, including photosynthesis^16^, energy metabolism, plant water, nutrient uptake imbalance, protein, etc.^17^, and ultimately affect the growth and yield of crops^18^. Osmotic stress in plants changes many enzymatic antioxidants, such as CAT, SOD, GOPX^19^, non-enzymatic antioxidants, such as carotenoids^20^, pigments like betalains, and chlorophylls, beta-carotene^21^, phenolic and flavonoids, etc. having high antiradical capacities^22^ which can manage the adverse impact of abiotic stresses. Under abiotic stress, the plant itself regulates different pathways to increase the application of these antioxidants to detoxify the ROS and mitigate environmental stress^23^. Therefore, the changes in physiological and biochemical indices and the antioxidant system of plants under drought stress are important manifestations to measure the differences in drought tolerance of plants. Flavonoids are important secondary metabolites that are distributed in various parts of plants. They are not only auxiliary color substances of plant flowers but also help plants stabilize oxidative homeostasis and resist osmotic stress, and metal ions combine to reduce damage to the cytoplasm to improve plant drought tolerance^24,25^.

Currently, research on the drought tolerance of chestnut has mainly focused on photosynthesis, membrane permeability, enzyme activity, osmotic substances and other physiological indicators^26–28^. Flavonoids and flavonols, as important antioxidants, are rarely used as drought tolerance indicators for evaluation^29^. In this paper, flavonoids and flavonols were involved in the ranking of drought tolerance of 6 chestnut varieties (Clones). Using the method of natural drought stress, the changes of 15 physiological and biochemical indexes of 6 chestnut varieties (Clones) under drought stress were studied. According to each index, the drought tolerance of 6 chestnut varieties (Clones) was comprehensively evaluated by principal component analysis and membership function method, which was the theoretical basis for the subsequent drought tolerance research of chestnut.

## Results

### Determination of the soil water content of different chestnut varieties (Clones) under drought stress

Table 1 showed that when the soil relative water content (RWC) of each test variety (clones) was approximately 30%, the sample leaves were collected. For each variety, the measurement was repeated three times to measure the soil relative water content and soil absolute water content, which were at the level of severe drought. It can be concluded that the absolute soil water content obtained by the aluminum box weighing method was significantly correlated with the relative soil water content measured by the instrument in Table 2.

**Table 1:** Determination of soil water content under drought stress.

Chestnuts varieties (clones)	Wet weight/g	Dry soil weight/g	Absolute soil water content %	Soil relative water content %
QL45	3.27	24.47	13.28aA	29.97aA
QX42	3.00	22.80	13.19aA	31.63aA
YSZF	3.10	22.17	14.04aA	30.40aA
YZ	3.13	23.83	13.10aA	30.50aA
YQ	3.13	23.13	13.54aA	31.90aA
YL	3.27	25.60	12.75aA	30.20aA

Different lowercase letters in the same trade indicate significant differences (P < 0.05). There were significant differences among different capital letters (P < 0.01).

**Table 2:** Correlation between soil water content and soil relative water content.

		Soil relative water content %
Absolute soil water content %	Pearson correlation	0.573*
	Sig. (double-tailed)	0.013
	Number of cases	18

*Indicating a significant correlation between the two.

### Analysis of enzyme system related indices of different chestnut varieties (clones) under drought stress

SOD scavenges harmful substances in the metabolic activities of fruit trees, scavenges free radicals produced by chestnut under drought stress, and repairs cells. It was observed that there was no significant difference between QX42, YQ, QL45, YSZF and YZ, and there was no significant difference between YSZF, YZ and YL. QX42, YQ, QL45, YSZF and YZ were significantly higher than YL in Table 3. Among them, the SOD activity of QX42 was the highest. Thus, QX42 had strong drought tolerance, followed by YQ and QL45.

**Table 3:** Analysis of enzyme related indexes of different chestnut varieties ( lines ) under drought stress.

Varieties (clones)	X_1_	X_2_	X_3_	X_4_
QX42	538.46aA	6105.00cC	1382.25aA	50.16abA
QL45	523.87aA	9861.5bB	1359.95aA	68.92aA
YSZF	468.51abA	9626.9bB	762.73cBC	53.14abA
YZ	422.42abA	15,369.68aA	1087.5bBC	58.90abA
YQ	531.01aA	15,726.23aA	662.72cdC	53.29abA
YL	344.20bA	13,751.13aA	495.8dC	41.72bA

X_1_-SOD activity (U/g FW), X_2_-POD activity (U/g FW), X_3_-CAT activity (nmol/min/g FW), X_4_-APX activity (μmol/min/g FW), the following table was the same.

Different lowercase letters in the same trade indicate significant differences (P < 0.05), while uppercase letters in the same trade indicate extremely significant differences (P < 0.01).

POD catalyzes many biochemical reactions in fruit trees and has the effect of eliminating cytotoxicity. Table 3 showed that under drought stress, the POD differences of different chestnut varieties showed that YQ, YZ and YL were significantly higher than QL45 and YSZF, and the latter two varieties were significantly higher than QX42. Under drought stress, the activities of YQ and YZ were higher than those of YL. The POD content of YQ was 2.58 times that of QX42.

CAT can decompose hydrogen peroxide into oxygen and water to prevent excessive damage to the cell membrane of fruit tree cells under drought stress. Table 3 shows that the difference between QX 42 and QL 45 was not significant, QX 42 and QL 45 were significantly higher than YZ, YSZF, YQ and YL and YZ, YZ and YL, QX 42 and QX 42, followed by QL 45, in which YL was the lowest and all other varieties were higher than the control YL.

APX is an important enzyme for fruit trees to resist oxidative stress, which helps remove excessive peroxides when fruit trees are subjected to drought stress. There was no significant difference between QL45, YZ, YQ, YSZF and QX42, and QL45 was significantly higher than YL. The activity of QL45 APX was 68.92 μmol/min/g fresh weight, 1.65 times that of YL, as shown in Table 3.

### Analysis of cell regulatory substance indices of different chestnut varieties (clones) under drought stress

The accumulation of betaine was slower in plants under drought stress. There was no significant difference in betaine content among YZ, YQ and YSZF, and there was no significant difference among YSZF, QX42 and QL45. The difference in betaine content between YZ and YQ was significantly higher than that between QX42 and QL45, and the lowest content was observed for YL. The betaine content in YZ was 4.41 times that in YL, as shown in Table 4.

**Table 4:** Analysis of cell material index under drought stress.

Varieties (clones)	X_5_	X_6_	X_7_	X_8_	X_9_	X_10_	X_11_
QX42	2.08cAB	4.75bcB	40.64dC	4.55deCD	2.11cA	232.04bB	17,914.23bB
QL45	2.24bcA	4.70bcB	49.24bcAB	4.71cdBCD	2.39abA	337.70aA	17,500.54bB
YSZF	2.63abcA	4.99abAB	43.85dBC	5.02bB	2.05cA	244.38bB	15,474.93bB
YZ	3.22aA	4.57cB	52.97abA	4.35eD	2.14bcA	232.86bB	6414.16cC
YQ	3.08abA	5.27aA	55.70aA	4.89bcBC	2.12cA	247.50bB	23,596.79aA
YL	0.73dB	4.04dC	45.54cdBC	6.10aA	2.40aA	229.16bB	16,718.33bB

X_5_—betaine content (mg/g dry weight), X_6_—soluble sugar content (X %), X_7_—soluble protein content (mg/g FW), X_8_—PRO content (μg/g FW), X_9_—chlorophyll content (mg/g), X_10_—Flavonoids content (ng/g), X_11_—Flavonol content (μg/g), the following table was the same.

Different lowercase letters in the same trade indicate significant differences (P < 0.05), while uppercase letters in the same trade indicate extremely significant differences (P < 0.01).

Soluble sugar is an important regulator of cell permeability. After drought stress treatment, the soluble sugar content of each chestnut variety (clone) was different. Table4 showed that the soluble sugar content of YQ and YSZF was significantly higher than that of QX42, QL45, YZ and YL. The difference in soluble sugar content of QX42 and QL45 was not significant, and the lowest soluble content was observed in YL.

Soluble proteins can dissolve in water, increase the water retention ability of fruit tree cells, and protect fruit trees from drought stress. Table 4 showed that YQ and YZ were significantly higher than QL45 and YL, and the latter two were significantly higher than YSZF and QX42. YQ, YZ, and QL45 were higher than YL, and YSZF and QX42 were lower than YL.

PRO is a common affinity osmoregulatory substance with no toxic effect. Under drought stress, intracellular regulatory substances actively accumulate, and the greater is the accumulation, the stronger is the drought tolerance. Table 4 showed that YL was significantly higher than the other varieties in PRO, and there was little difference between YSZF and YQ. Among them, the highest PRO content was observed in YL, and YZ was the cultivar with the lowest PRO content. It has a certain drought tolerance ability under drought stress, but the degree is different.

Chlorophyll is the main pigment for photosynthesis and can help plants store nutrients. There was no significant difference in chlorophyll content among YZ, YQ, QX42 and YSZF. The chlorophyll content of YL was significantly higher than that of YZ, YQ, QX42 and YSZF. The chlorophyll content of QL45 and YL was significantly higher than that of YZ, YQ and YSZF, as shown in Table 4. The highest chlorophyll content of YL was 2.40 mg/g, 1.17 times that of YSZF, as shown in Table 4.

Flavonoids have a strong oxidation effect and can remove too many oxygen free radicals. The flavonoid content of QL45 leaves was significantly higher than that of QX42, YSZF, YZ, YQ and YL, as shown in Table 4. The content of flavonoids in QL45 was 337.70 mg/g, which was 1.47 times that of YL. In Table 4, the flavonoid content of QL45 was significantly higher than the other five varieties (clones).

Flavonol was a kind of substance with large quantity and range in flavonoids, which can inhibit stomatal closure of plants. As shown in Table 4, the flavonoid content of YQ leaves was significantly higher than that of QX42, YSZF, YZ, YQ and YL. The content of flavonoids in YQ was 23,596.79ng/g, which was 1.47 times that of YL.

### Analysis of harmful substance content in different chestnut varieties (clones) under drought stress

MDA is an important product of cell membrane lipid peroxidation, which can combine with proteins and enzymes on the cell membrane and destroy the cell membrane structure. The level of cell membrane lipid peroxidation can be reflected by the MDA content. Therefore, MDA content can be used as one of the detection indices of fruit trees subjected to stress damage. Table 4 showed that the MDA content of each chestnut variety was different under drought stress. The difference between YSZF, YZ and QL45 was not significant, and the difference between QL45, YQ, QX42 and YL was not significant. The MDA content of YSZF and YZ was significantly higher than that of YQ, QX42 and YL. The MDA contents of the six varieties (clones) of chestnut were YSZF, YZ, QL45, YQ, QX42 and YL from high to low, respectively. The MDA content of YSZF was 1.25 times that of YL (Table 5). Compared with the control group, these values were higher than that of YL, indicating that drought stress caused varying degrees of membrane lipid peroxidation, resulting in MDA accumulation.

**Table 5:** Analysis of harmful substance content under drought stress.

Varieties (clones)	X_12_	X_13_	X_14_	X_15_
QX42	112.55cA	257.37aA	12.87aA	36.66aA
QL45	123.93abcA	115.11cC	5.76cC	37.56aA
YSZF	140.12aA	130.21bcC	6.51bcC	27.48cAB
YZ	135.40abA	177.34bBC	8.87bBC	30.20bcAB
YQ	117.88bcA	242.15aAB	12.11aAB	24.56cB
YL	111.99cA	159.13bcC	7.96bcC	35.24abA

X_12_—MDA content (nmol/g), X_13_—OFR content, X_14_—OFR production rate (nmol/g min), X_15_—H_2_O_2_ content, The following table was the same.

Different lowercase letters in the same trade indicate significant differences (P < 0.05), while uppercase letters in the same trade indicate extremely significant differences (P < 0.01).

Table 5 showed that compared with the OFR content, QX42 and YQ were significantly higher than YZ, YL and YSZF, and the latter three were significantly higher than QL45. Under normal conditions, the content was low and will not cause damage to cells. Under drought stress, it will accumulate too much and cause toxicity to cells. The OFR contents in leaves of six varieties (clones) from high to low were QX42, YQ, YZ, YL, YSZF and QL45, respectively.

The OFR production rate is too fast, which will cause an imbalance in reactive oxygen species production and removal in fruit tree cells. There was no significant difference in the OFR production rate between QX42 and YQ and no significant difference between YZ, YL, YSZF and QL45. QL45 was lower than other varieties (clones) (Table 5).

The H_2_O_2_ content is an important representative of the reactive oxygen species content, and the production and removal are balanced. Under drought stress, fruit tree cells produce a large amount of H_2_O_2_ to destroy the cell membrane, which reduces the yield of fruit trees. Table 5 showed that the difference in H_2_O_2_ content between QL45, QX42, QL and YZ was not significant, the difference in H_2_O_2_ content between YZ, YSZF and YQ was not significant, and the difference in QL45, QX42, QL and YL was significantly higher than that in YZ, YSZF and YQ.

### Comparison of drought tolerance-related indices reflecting the drought tolerance of different chestnut varieties (clones)

The correlation coefficient matrix was obtained by analyzing the correlation of drought tolerance indices of six chestnut varieties (clones) (Table 6). The correlation coefficient matrix showed that there is a certain correlation between each drought tolerance index pair, and the information reflected by the data overlaps to a certain extent. The direct use of these indices cannot accurately evaluate the drought tolerance of chestnut. Through principal component analysis, the drought tolerance evaluation indices can be better selected by comparing the drought tolerance ability of each index. The principal component comprehensive evaluation can comprehensively and reasonably analyze the dominant factors in many factors, and the evaluation results are highly reliable. According to the principle that the cumulative contribution rate of principal component analysis was greater than 80%^30^, the first four principal components were extracted as comprehensive indices for drought tolerance evaluation of chestnut (Table 7). The first principal component contribution rate was 32.36%, the second principal component contribution rate was 24.88%, the third principal component contribution rate was 21.11%, and the fourth principal component contribution rate was 12.88%. The first four principal components reflected 91.24% of the information related to drought tolerance. SOD, betaine, soluble sugars, chlorophyll, and PRO had higher loads in the first principal component, indicating that the first principal component mainly reflected the relationship between these indicators and drought tolerance. CAT activity, APX activity, OFR content, OFR production rate, and flavone content had higher loads on the second principal component, indicating that the second principal component mainly reflected the relationship between these indices and drought tolerance. And MDA content, POD activity, soluble protein content, and H_2_O_2_ content had higher loads on the third principal component, indicating that the third principal component mainly reflected the relationship between these indicators and drought tolerance. The flavonol content in the fourth principal component has a high load, indicating that the fourth principal component mainly reflects the relationship between flavonol content and drought tolerance.

**Table 6:** Correlation coefficient matrix of 15 indicators.

	X_1_	X_2_	X_3_	X_4_	X_5_	X_6_	X_7_	X_8_	X_9_	X_10_	X_11_	X_12_	X_13_	X_14_	X_15_
X_1_	1														
X_2_	− 0.455	1													
X_3_	0.593	− 0.613	1												
X_4_	0.519	− 0.083	0.652	1											
X_5_	0.527	0.229	0.257	0.557	1										
X_6_	0.797*	− 0.053	0.109	0.349	0.770*	1									
X_7_	0.051	0.842*	− 0.216	0.384	0.567	0.32	1								
X_8_	− 0.676	0.208	− 0.756*	− 0.681	− 0.816*	− 0.556	− 0.222	1							
X_9_	− 0.391	0.114	− 0.032	0.088	− 0.686	− 0.688	0.026	0.532	1						
X_10_	0.423	− 0.208	0.474	0.817*	0.059	0.134	0.178	− 0.213	0.507	1					
X_11_	0.486	− 0.146	− 0.221	− 0.182	− 0.189	0.433	− 0.006	0.289	0.070	0.193	1				
X_12_	− 0.047	0.104	0.036	0.441	0.607	0.289	0.167	− 0.399	− 0.441	0.076	− 0.587	1			
X_13_	0.377	− 0.011	0.068	− 0.387	0.187	0.322	0.044	− 0.25	− 0.468	− 0.534	0.344	− 0.534	1		
X_14_	0.377	− 0.011	0.068	− 0.386	0.187	0.322	0.044	− 0.25	− 0.467	− 0.533	0.344	− 0.534	1.000**	1	
X_15_	− 0.073	− 0.558	0.559	0.106	− 0.639	− 0.652	− 0.544	0.105	0.655	0.371	− 0.116	− 0.445	− 0.193	− 0.193	1

X_1_—SOD activity (U/g FW), X_2_—POD activity (U/g FW), X_3_—CAT activity (nmol/min/g FW), X_4_—APX activity(μmol/min/g FW), X_5_—betaine content (mg/g dry weight), X_6_—soluble sugar content (X %), X_7_—soluble protein content (mg/g FW), X_8_—PRO content (μg/g FW), X_9_—chlorophyll content (mg/g), X_10_—Flavonoids content (mg/g), X_11_—Flavonol content (ng/g), X_12_—MDA content (nmol/g), X_13_—OFR content, X_14_—OFR production rate (nmol/g min), X_15_—H_2_O_2_ content.

*Indicates significant difference at 0.05 level; **indicates extremely significant difference at 0.01 level.

**Table 7:** Principal component coefficient, eigenvalue and contribution rate.

Principal components	X_1_	X_2_	X_3_	X_4_	X_5_	X_6_	X_7_	X_8_	X_9_	X_10_	X_11_	X_12_	X_13_	X_14_	X_15_	Eigen value	Contribution rate (%)	Cumulative contribution rate (%)
1	0.775	− 0.035	0.422	0.569	0.942	0.878	0.397	− 0.873	− 0.705	0.150	0.037	0.427	0.325	0.325	− 0.506	4.855	32.364	32.364
2	0.130	− 0.360	0.617	0.773	− 0.016	− 0.191	− 0.110	− 0.275	0.456	0.822	− 0.326	0.337	− 0.726	− 0.726	0.569	3.732	24.883	57.247
3	0.538	− 0.765	0.545	− 0.107	− 0.317	0.040	− 0.591	− 0.136	0.024	0.089	0.463	− 0.662	0.508	0.508	0.581	3.167	21.115	78.362
4	0.257	0.364	− 0.221	0.221	− 0.061	0.230	0.556	0.243	0.481	0.535	0.706	− 0.432	− 0.002	− 0.002	− 0.092	1.932	12.879	91.241

The corresponding coefficients of each index in the four principal components are obtained by dividing the corresponding data by the square root of the corresponding eigenvalue of the principal component, and the four principal component expressions F_1_, F_2_, F_3_ and F_4_ are obtained. Then, the principal component comprehensive model was obtained by taking the proportion of the corresponding eigenvalue of each principal component in the total feature and the proportion of the extracted principal component as the weight^31^:

$${\text{F}} = \lambda_{1} /(\lambda_{1} + \lambda_{2} + \lambda_{3} + \lambda_{4} )F_{1} + \lambda_{2} /(\lambda_{1} + \lambda_{2} + \lambda_{3} + \lambda_{4} )F_{2} + \lambda_{3} /(\lambda_{1} + \lambda_{2} + \lambda_{3} + \lambda_{4} )F_{3} + \lambda_{4} /(\lambda_{1} + \lambda_{2} + \lambda_{3} + \lambda_{4} )F_{4} .$$

Specifically:

$$\begin{gathered} {\text{F}}_{{1}} = \, 0.{\text{352X}}_{{1}} - \, 0.0{\text{16X}}_{{2}} + \, 0.{\text{192X}}_{{3}} + \, 0.{\text{258X}}_{{4}} + \, 0.{\text{428X}}_{{5}} + \, 0.{\text{399X}}_{{6}} + \, 0.{18}0{\text{X}}_{{7}} \hfill \\ - 0.{\text{396X}}_{{8}} - \, 0.{32}0{\text{X}}_{{9}} + \, 0.0{\text{68X}}_{{{1}0}} + \, 0.0{\text{17X}}_{{{11}}} + \, 0.{\text{194X}}_{{{12}}} + \, 0.{\text{148X}}_{{{13}}} + \, 0.{\text{148X}}_{{{14}}} - \, 0.{23}0{\text{X}}_{{{15}}} \hfill \\ {\text{F}}_{{2}} = \, 0.0{\text{67X}}_{{1}} - \, 0.{\text{186X}}_{{2}} + \, 0.{\text{319X}}_{{3}} + \, 0.{4}00{\text{X}}_{{4}} - \, 0.00{\text{8X}}_{{5}} - \, 0.0{\text{99X}}_{{6}} - \, 0.0{\text{57X}}_{{7}} \hfill \\ - 0.{\text{142X}}_{{8}} + \, 0.{\text{236X}}_{{9}} + \, 0.{\text{425X}}_{{{1}0}} - \, 0.{\text{169X}}_{{{11}}} + \, 0.{\text{174X}}_{{{12}}} - \, 0.{\text{376X}}_{{{13}}} - \, 0.{\text{376X}}_{{{14}}} + \, 0.{\text{295X}}_{{{15}}} \hfill \\ {\text{F}}_{{3}} = \, 0.{3}0{\text{2X}}_{{1}} - \, 0.{43}0{\text{X}}_{{2}} + \, 0.{3}0{\text{6X}}_{{3}} - \, 0.0{6}0{\text{X}}_{{4}} - \, 0.{\text{178X}}_{{5}} + \, 0.0{\text{22X}}_{{6}} - \, 0.{\text{332X}}_{{7}} \hfill \\ - 0.0{\text{76X}}_{{8}} + \, 0.0{\text{13X}}_{{9}} + \, 0.0{5}0{\text{X}}_{{{1}0}} + \, 0.{26}0{\text{X}}_{{{11}}} - \, 0.{\text{372X}}_{{{12}}} + \, 0.{\text{285X}}_{{{13}}} + \, 0.{\text{285X}}_{{{14}}} + \, 0.{\text{326X}}_{{{15}}} \hfill \\ {\text{F}}_{{4}} = \, 0.{\text{185X}}_{{1}} + \, 0.{\text{262X}}_{{2}} - \, 0.{\text{159X}}_{{3}} + \, 0.{\text{159X}}_{{4}} - \, 0.0{\text{44X}}_{{5}} + \, 0.{\text{165X}}_{{6}} + \, 0.{4}00{\text{X}}_{{7}} \hfill \\ + \, 0.{\text{175X}}_{{8}} + \, 0.{\text{346X}}_{{9}} + \, 0.{\text{385X}}_{{{1}0}} + \, 0.{5}0{\text{8X}}_{{{11}}} - \, 0.{\text{311X}}_{{{12}}} - \, 0.00{\text{1X}}_{{{13}}} - \, 0.00{\text{1X}}_{{{14}}} - \, 0.0{\text{66X}}_{{{15}}} \hfill \\ \end{gathered}$$

The principal component synthesis model was as follows:

$$\begin{gathered} {\text{F }} = \, 0.{\text{239X}}_{{1}} - \, 0.{\text{119X}}_{{2}} + \, 0.{2}0{\text{4X}}_{{3}} + \, 0.{2}0{\text{9X}}_{{4}} + \, 0.{1}0{\text{2X}}_{{5}} + \, 0.{\text{143X}}_{{6}} + \, 0.0{\text{28X}}_{{7}} \hfill \\ - 0.{\text{172X}}_{{8}} + \, 0.00{\text{3X}}_{{9}} + \, 0.{2}0{\text{6X}}_{{{1}0}} + \, 0.0{\text{92X}}_{{{11}}} - \, 0.0{\text{13X}}_{{{12}}} + \, 0.0{\text{16X}}_{{{13}}} + \, 0.0{\text{16X}}_{{{14}}} + \, 0.0{\text{65X}}_{{{15}}} \hfill \\ \end{gathered}$$

The greater is the weight of each index, the greater is the importance, and vice versa. Therefore, among the drought tolerance indices of chestnut, the order of drought tolerance ability from high to low was as follows: SOD activity, APX activity, flavone content, CAT activity, PRO content, soluble sugar content, POD activity, betaine content, flavonol content, H_2_O_2_ content, protein content, soluble protein content, OFR content, OFR production rate, MDA content, chlorophyll content.

### Comprehensive evaluation of drought tolerance (membership function method)

The membership function values of different chestnuts were obtained by the membership function method; then, the drought tolerance measurement values were obtained by combining the weight of each index (Table 8) to sort the drought tolerance. The greater is the drought tolerance measure, the stronger is the drought tolerance. Table 7 shows that the maximum drought tolerance was found in QL45 (1.333), and the minimum was found in YL (0.168). The average membership functions of 14 physiological indices in the leaves of 6 varieties (clones), i.e., QX42, QL45, YSZF, YZ, YQ and YL, were determined 1.090, 1.333, 0.773, 0.780, 0.910, 0.168 (Table 7). Based on this, the drought tolerance of six chestnut varieties (clones) was ranked, and the drought tolerance from high to low was as follows: QL45, QX42, YQ, YZ, YSZF, YL.

**Table 8:** 6 subordinate function values, weights and orders of drought tolerance of chestnuts.

Varieties (clones)	R_1_	R_2_	R_3_	R_4_	R_5_	R_6_	R_7_	R_8_	R_9_	R_10_	R_11_	R_12_	R_13_	R_14_	R_15_	Drought-tolerance value	order
QX42	1.000	1.000	1.000	0.310	0.542	0.577	0.000	0.886	0.171	0.027	0.669	0.980	1.000	1.000	0.931	1.090	2
QL45	0.925	0.610	0.975	1.000	0.606	0.537	0.571	0.794	0.971	1.000	0.645	0.576	0.000	0.000	1.000	1.333	1
YSZF	0.640	0.634	0.301	0.420	0.763	0.772	0.213	0.617	0.000	0.140	0.527	0.000	0.106	0.105	0.225	0.773	5
YZ	0.403	0.037	0.667	0.632	1.000	0.431	0.819	1.000	0.257	0.034	0.000	0.168	0.437	0.437	0.434	0.780	4
YQ	0.962	0.000	0.188	0.426	0.944	1.000	1.000	0.691	0.200	0.169	1.000	0.791	0.893	0.893	0.000	0.910	3
YL	0.000	0.205	0.000	0.000	0.000	0.000	0.325	0.000	1.000	0.000	0.600	1.000	0.309	0.309	0.822	0.168	6

## Discussion

Drought stress creates reactive oxygen species which hampers the water retention of cells, causes cell swelling and destruction of the cell membrane^19,32^. Drought tolerance was evaluated by comprehensive comparison of physiological and biochemical indices among different chestnut varieties (clones). As a type of drought-resistant fruit tree, it is of great significance to identify the drought tolerance of chestnut for drought-resistant breeding and breeding varieties suitable for cultivation. There are many studies on chestnut enzyme activity, chlorophyll, membrane permeability, osmotic regulation and MDA under drought stress. *Castanea mollissima* was subjected to drought stress, and MDA and osmotic adjustment substances were increased, indicating that drought stress led to different varieties (clones) of *Castanea mollissima* to experience varying degrees of membrane damage as well as water loss stress. In addition, a positive response to drought stress was observed, this is consistent with the results of previous studies on chestnut^31^, lily^33^ and apricot^34^. The study of active oxygen protective enzymes in several chestnut varieties (clones) showed that SOD, POD, CAT and APX increased synchronously under drought stress, which indicated that they had synergistic effect, which was also consistent with the results of previous studies^26,35^. The soluble sugar content of different chestnut varieties (clones) under drought stress was different, and the content was higher than that of the YL of rootstock varieties.

There are various ways to evaluate the drought response of plants, and the genes that control the physiological response of plants to drought are also multiple genes. Different plants have different drought tolerance mechanisms, and the drought tolerance of the same plant is also different. Therefore, it is insufficient to evaluate the drought of plants by a single indicator, and there are certain limitations^10–15^; thus, multiple indicators should be integrated^36^. In this experiment, grafting trees of six chestnut varieties (clones) were used as experimental materials, and 15 drought tolerance indices were selected for correlation analysis. Principal component analysis was performed to determine the index weight. The component model shows that the drought tolerance indices of chestnut from high to low are as follows: SOD activity, APX activity, flavone content, CAT activity, PRO content, soluble sugar content, POD activity, betaine content, flavonol content, H2O2 content, protein content, soluble protein content, OFR content, OFR production rate, MDA content, chlorophyll content. Based on the membership function value, 15 physiological and biochemical indices were comprehensively analyzed by the principal component evaluation method. The obtained results showed that the drought tolerance of the 6 different varieties (clones) of chestnut was QL45 > QX42 > YQ > YZ > YSZF > YL. In the study of Guo Yan et al., YSZF and YQ drought tolerance belong to medium drought tolerance varieties. In this study, the drought tolerance of the same two varieties was not much different^37^. In addition, Wu and Guo et al. used potted seedlings and roots as the main indicators of comprehensive evaluation, and found that the drought tolerance of YL was greater than that of YSZF, which was contrary to the results of this study^31^. Growth states and index comprehensive evaluation results will be different, and twe will be a follow-up study on potted seedlings of chestnut with drought tolerance.

Multivariate statistics based on multiple indicators can accurately reflect the drought tolerance of different chestnut varieties (clones), providing a theoretical basis for the study of drought tolerance in chestnut varieties (clones).

## Conclusion

In this study, principal component analysis and membership function method were used to study the drought tolerance of six chestnut varieties (Clones) based on different physiological indexes and two important secondary metabolites of flavonoids and flavonols as evaluation indexes. The results showed that flavonoids and flavonols ranked top among 15 tolerance -related indicators and could be used as tolerance indicators. The drought tolerance of 6 chestnut varieties (clones) was : QL45 > QX42 > YQ > YZ > YSZF > YL.

## Materials and methods

### Location

Hebei Normal University of Science and Technology Changli Shigezhuang Chestnut Germplasm Resources Nursery is located in the northeast of Hebei Province, southwest of Qinhuangdao. It is between 39° 22′ and 39° 48′ N and 118°45′-119°20′E and has the warm temperate semihumid continental monsoon climate in the eastern monsoon region of China. The annual average temperature is 11.8 °C, and the annual precipitation is 527.0 mm. The annual sunshine is 2719.5 h, annual sunshine percentage is 61%, frost-free period is 210 days, maximum frozen depth is 53 cm, and icing is 117 days.

### Experimental design

The grafting trees of six chestnut varieties (clones) were Qianxi 42 (QX42), Qinglong 45 (QL45), Yanshan Zaofeng (YSZF), Yanzi (YZ), Yanqiu (YQ) and Yanlong (YL). In April 2019, 6 varieties (clones) were grafted onto rootstocks of the 5-year-old chestnut cultivar YL at the Changli Shigezhuang base. Each variety (clones) grafted 5 trees. In May 2021, 6 grafting trees of chestnut varieties (clones) in the resource nursery were selected as experimental materials. Unified conventional cultivation management was implemented, and a randomized block test was performed. Each variety (clones) was used to establish a single plant plot, which was repeated three times. In July 2021, the natural drought stress test was performed by watering one time. When the soil relative water content (RWC) decreased to approximately 30%, leaf samples were collected to determine the physiological and biochemical indices related to drought tolerance (the relative water content of agricultural drought grade standard soil was approximately 30%-40% for severe drought^38^.

### Determination method

Three grafting trees of six chestnut varieties (clones) with basically the same growth management were selected to determine the soil relative water content. When the soil RWC was reduced to approximately 30%, mature leaves with similar sizes on the eastern main branch of chestnut varieties (clones) were collected on July 23, 2021, from 8:00 to 10:00 in the morning. Ten leaves were collected from each tree, and a total of 30 leaves were collected from one variety (clones). The labeled leaves were placed in an ice box and brought back to the laboratory. After liquid nitrogen treatment, they were stored in an ultra-low temperature refrigerator for testing.

### Etermination of soil water content

The absolute soil water content was determined by drying and weighing method^21^, and the soil relative water content was measured by the JK-300F soil moisture meter of Xinghua Youke Instrument Co., Ltd.

### Determination of physiological and biochemical indicators

The MDA content was determined with reference to thiobarbituric acid colorimetry; the activity of SOD was determined by reference to the nitroblue tetrazolium photoreduction method; POD activity was determined according to the guaiacol colorimetric method^39^. the chlorophyll content^40^, PRO content^41^, H_2_O_2_ content^41^, APX activity^41^ and CAT activity^39^ were determined by spectrophotometry; The soluble sugar content was determined by anthrone colorimetry. Soluble protein content was determined by coomassie brilliant blue method; the OFR content^40^ and OFR production rate^41^ was determined by the hydroxyl ammonia oxidation method; The betaine content was determined by chemical colorimetry^41^. The content of flavonoids and flavonol were determined by HPLC^42^; A single index cannot effectively evaluate the drought tolerance of chestnut. In this study, 15 physiological and biochemical indices related to drought tolerance were selected to comprehensively evaluate the drought tolerance of chestnut^43^. All of these indexes were measured by using the kit, which came from Suzhou Keming Biotechnology Co., LTD. in China.

### Data analysis

Microsoft Excel 2010 was used for data integration. SPSS 26 and DPS 9.01 software were used for analysis of variance and principal component analysis. The membership function method was used to comprehensively evaluate the drought tolerance of chestnut. The related membership function values of each index and drought tolerance were calculated.

### Membership function method

The calculation formula of the membership function value of each index and drought tolerance is as follows. When the index is positively correlated with drought tolerance, the calculation result is expressed as R (X_ij_) = (X_ij_-X_min_)/(X_max_-X_min_). When the index is negatively correlated with drought tolerance, the calculation results are expressed as R (X_ij_) = 1 − (X_ij_ − X_min_)/(X_max_ − X_min_), where R (X_ij_) is the drought tolerance membership function value of i variety j, X_ij_ is the measured value of j index of i variety, and xmax and Xmin are the maximum and minimum values of j index. According to the membership function value and the weight of each index, the drought tolerance measurement value was calculated according to the membership function value and the weight of each index. The greater is the drought tolerance measure, the stronger is the drought tolerance ability. The calculation formula of drought tolerance (D) is as follows^31^:

$$\text{D}={\sum }_{j=1}^{m}(R({X}_{ij}){*W}_{j})$$

In the formula, W_j_ was the index weight.

### Ethics statement

The experiment is in line with relevant institutions, national and international norms and legislation.

## Data Availability

The datasets used and/or analysed during the current study available from the corresponding author on reasonable request.
